# Do green spaces affect the spatiotemporal changes of PM_2.5_ in Nanjing?

**DOI:** 10.1186/s13717-016-0052-6

**Published:** 2016-05-25

**Authors:** Jiquan Chen, Liuyan Zhu, Peilei Fan, Li Tian, Raffaele Lafortezza

**Affiliations:** 1 International Center for Ecology, Meteorology, and Environment, Nanjing University of Information Science and Technology, Nanjing, 210044 China; 2 CGCEO/Geography, Michigan State University, East Lansing, MI 48824 USA; 3 School of Planning, Design, and Construction, Michigan State University, East Lansing, MI 48824 USA; 4 Institute of Geographic Sciences and Natural Resources Research, Chinese Academy of Sciences, Beijing, 100101 China; 5 Department of Scienze Agro-Ambientali e Territoriali, University of Bari, Bari, 70126 Italy; 6 Department of Geography, Michigan State University, 1405 S. Harrison Road, East Lansing, MI 48823 USA

**Keywords:** PM_2.5_, Green space, Edge density, Nanjing, Pollution control, Seasonal variation, Nature-Based Solution (NBS)

## Abstract

**Introduction:**

Among the most dangerous pollutants is PM_2.5_, which can directly pass through human lungs and move into the blood system. The use of nature-based solutions, such as increased vegetation cover in an urban landscape, is one of the possible solutions for reducing PM_2.5_ concentration. Our study objective was to understand the importance of green spaces in pollution reduction.

**Methods:**

Daily PM_2.5_ concentrations were manually collected at nine monitoring stations in Nanjing over a 534-day period from the air quality report of the China National Environmental Monitoring Center (CNEMC) to quantify the spatiotemporal change of PM_2.5_ concentration and its empirical relationship with vegetation and landscape structure in Nanjing.

**Results:**

The daily average, minimum, and maximum PM_2.5_ concentrations from the nine stations were 74.0, 14.2, and 332.0 μg m^−3^, respectively. Out of the 534 days, the days recorded as “excellent” and “good” conditions were found mostly in the spring (30.7 %), autumn (25.6 %), and summer (24.5 %), with only 19.2 % of the days in the winter. High PM_2.5_ concentrations exceeding the safe standards of the CNEMC were recorded predominately during the winter (39.3–100.0 %). Our hypothesis that green vegetation had the potential to reduce PM_2.5_ concentration was accepted at specific seasons and scales. The PM_2.5_ concentration appeared very highly correlated (*R*^2^ > 0.85) with green cover in spring at 1–2 km scales, highly correlated (*R*^2^ > 0.6) in autumn and winter at 4 km scale, and moderately correlated in summer (*R*^2^ > 0.4) at 2-, 5-, and 6-km scales. However, a non-significant correlation between green cover and PM_2.5_ concentration was found when its level was >75 μg m^−3^. Across the Nanjing urban landscape, the east and southwest parts had high pollution levels.

**Conclusions:**

Although the empirical models seemed significant for spring only, one should not devalue the importance of green vegetation in other seasons because the regulations are often complicated by vegetation, meteorological conditions, and human activities.

## Introduction

Smog, also known as “smoke fog” (i.e., combination of smoke and other atmospheric pollutants), has become increasingly recognized as a primary environmental problem in many cities worldwide. The problem spread widely in European and North American cities in the 1950s and 1960s but has since become more pronounced in developing countries (e.g., India, China) (Rohde and Muller 2015; Zhang and Cao 2015). Among the most dangerous pollutants is particulate matter of 2.5 μm or less in diameter—PM_2.5_—as it can pass directly through human lungs and into the blood system. It is notorious for its role in increasing heart disease, stroke, emphysema, and lung cancer (Apte et al. 2015; Tong et al. 2015).

PM_2.5_ concentration is primarily determined by the emission source within an urban landscape and its area of reach (Querol et al. 2004; Rohde and Muller 2015). Heavy industries and the escalating number of vehicles, fugitive dust, and biomass combustions are the main causes of high pollutants throughout global cities (Rodrıguez et al. 2004; Giugliano et al. 2005; Park and Kim 2005; Viana et al. 2008; Mugica et al. 2009; Perrone et al. 2012). Atmospheric conditions such as vertical and horizontal temperature, atmosphere pressure, wind speed, and water vapor density are mostly responsible for PM_2.5_ dispersion (i.e., the sinks) (Liu 1985; Janhäll 2015; Rohde and Muller 2015). Consequently, the temporal changes in local and regional climate play fundamental roles in PM_2.5_ concentrations over hourly to interannual time scales (Hueglin et al. 2005; Vecchi et al. 2007), leading to high levels during the night (Vecchi et al. 2007) and in winter (Vecchi et al. 2004; Giugliano et al. 2005; Zhang and Cao 2015). Within the landscape, PM_2.5_ can be decomposed over time through multiple chemical, physical, and biological processes such as dry and wet deposition. Land surface properties, such as roads, construction, and vegetation, can directly filter pollutants and indirectly influence the air movement through its heterogeneous urban canopies (Janhäll 2015). While the ultimate solution for most cities is to eliminate the emission sources, other proposals to reduce PM_2.5_ concentration are also underway; these include engineering methods for filtration and uptake (Hänninen et al. 2005) or using nature-based solutions (NBS), such as increasing vegetation cover in urban landscapes.

Over the years, one of the most favored NBS to deal with urban problems, such as pollution reduction, has been to increase green vegetation. It has been theorized that green vegetation has the potential to reduce pollutants through filtration (Hwang et al. 2011) and the ability to regulate microclimatic conditions (Chen et al. 1999; Lafortezza et al. 2009). In a recent meta-analysis of 102 peer-reviewed publications on urban green space, urban heat islands, and air quality, Zupancic et al. (2015) stated that, “In general, the research suggests that balancing urban forest density, particularly in areas with low green space density, would greatly improve both local- and city-wide urban air quality.” The empirical and mechanistic functions of green spaces in reducing PM_2.5_ and other pollutants have also been reported in an increasing number of studies over recent decades. Based on a controlled experiment, Hwang et al. (2011) found that coniferous trees are more effective in filtering small particulates than broadleaf trees. Chen et al. (2015) demonstrated that PM_2.5_ inside forest shelterbelts is significantly higher than outside (i.e., captured pollutants), especially for larger particulates (e.g., PM_10_). At landscape level, Wu et al. (2015) found that the total vegetation cover and its spatial configuration (e.g., edge density, patch density, and aggregation) in Beijing could reduce annual PM_2.5_ concentration. Altogether, the roadside vegetation was credited for removing 1.09 Mg PM_2.5_ per year in Beijing (Tong et al. 2015). In the United States, Nowak et al. (2013) reported that, “The total amount of PM_2.5_ removed annually by trees varied from 4.7 t in Syracuse to 64.5 t in Atlanta, with annual values [of the trees in pollution reduction] varying from $1.1 million in Syracuse to $60.1 million in New York City.”

The PM_2.5_ concentration in an urban landscape is not static, but varies greatly in time and space (Querol et al. 2004; Nowak et al. 2013; Janhäll 2015) because of the dynamic meteorological conditions, heterogeneous land surface properties, uneven distribution of emission sources, landforms, and other human activities (e.g., vehicle use and biomass burning). Regardless of the large number of studies on vegetation and PM_2.5_ concentration, it is unclear if the role of vegetation in reducing PM_2.5_ concentration remains the same in different seasons and under varying pollution levels. This is especially critical for the cities in temperate zones where vegetation structure (e.g., amount and types of leaves) and composition (e.g., evergreen vs deciduous) are distinctively different among the four seasons. One would logically reason that the role of vegetation in filtering pollutants would be stronger with an elevated leaf surface, a higher leaf quantity, a more complex vertical canopy structure, a higher edge density, and numerously dispersed patch patterns (Janhäll 2015). These stand and landscape characteristics would raise a higher surface in three-dimensional space to capture more pollutants and would also promote a higher vapor density (Chen et al. 1999), which has the potential to reduce dust while increasing wet deposition. While some of these features do not change (e.g., land form), vegetation structure is highly dynamic, suggesting that the reduction will be highly dependent on seasons. Finally, the effect of a specific location in the landscape on PM_2.5_ depends on the surrounding vegetation (amount and configuration). Yet, we do not know the effective footprint of vegetation—i.e., the distance from the point of concern (e.g., emission source, high population concentration) where green space and landscape structure may have significant roles in reducing PM_2.5_ concentration.

A comprehensive examination on the spatiotemporal changes of PM_2.5_ in Nanjing was conducted based on the daily PM_2.5_ data collected from the China National Environmental Monitoring Center (CNEMC). Using this information, we explored the empirical relationships in PM_2.5_ concentrations with vegetation coverage and landscape characteristics to understand the importance of green vegetation in pollution reduction. The major hypothesis of this study was that vegetation and its spatial configuration play significant roles in reducing PM_2.5_ concentration in an urban landscape. These influences, however, varied by time (e.g., hours, days, months, seasons, weekdays), climatic conditions, and pollution levels (Zhang and Cao 2015). While it is well known that the atmospheric condition plays a major role in transporting (e.g., dispersion) and decomposing PM_2.5_ (Querol et al. 2004; Liu et al. 2005), the underlining mechanisms are complex and therefore beyond the scope of this study. Here, we focused on the empirical relationships between vegetation and the spatiotemporal changes of PM_2.5_. Our specific objectives were to (1) quantify the temporal changes in PM_2.5_ at daily, monthly, and seasonal scales, as well as the change during weekdays and weekends; (2) explore the spatial distribution and variations of PM_2.5_ across the Nanjing urban landscape; and (3) examine the empirical relationships between the spatiotemporal changes of PM_2.5_ and the distribution of green vegetation (forests, grasslands, lawns, crops, etc.). Our first premise is that PM_2.5_ concentration is not a constant because of (1) the dynamics and contrasting meteorological conditions, which determine the vertical and horizontal movement (including dispersion) of PM_2.5_ (e.g., seasonal changes) and (2) the different activities responsible for PM_2.5_ emission over time and space (e.g., oil/coal burning, automobile exhaustion, construction, emission from factories). This premise was further strengthened by the uneven distributions of green vegetation, which play significant roles in reducing PM_2.5_ but vary in regulatory reduction by time and location. While the landscape structure during the short study period remained unchanged, Nanjing’s location in the temperate zone suggests that the amount of green leaves as well as their functions in intercepting PM_2.5_ vary significantly by season.

## Methods

**Fig. 1:**
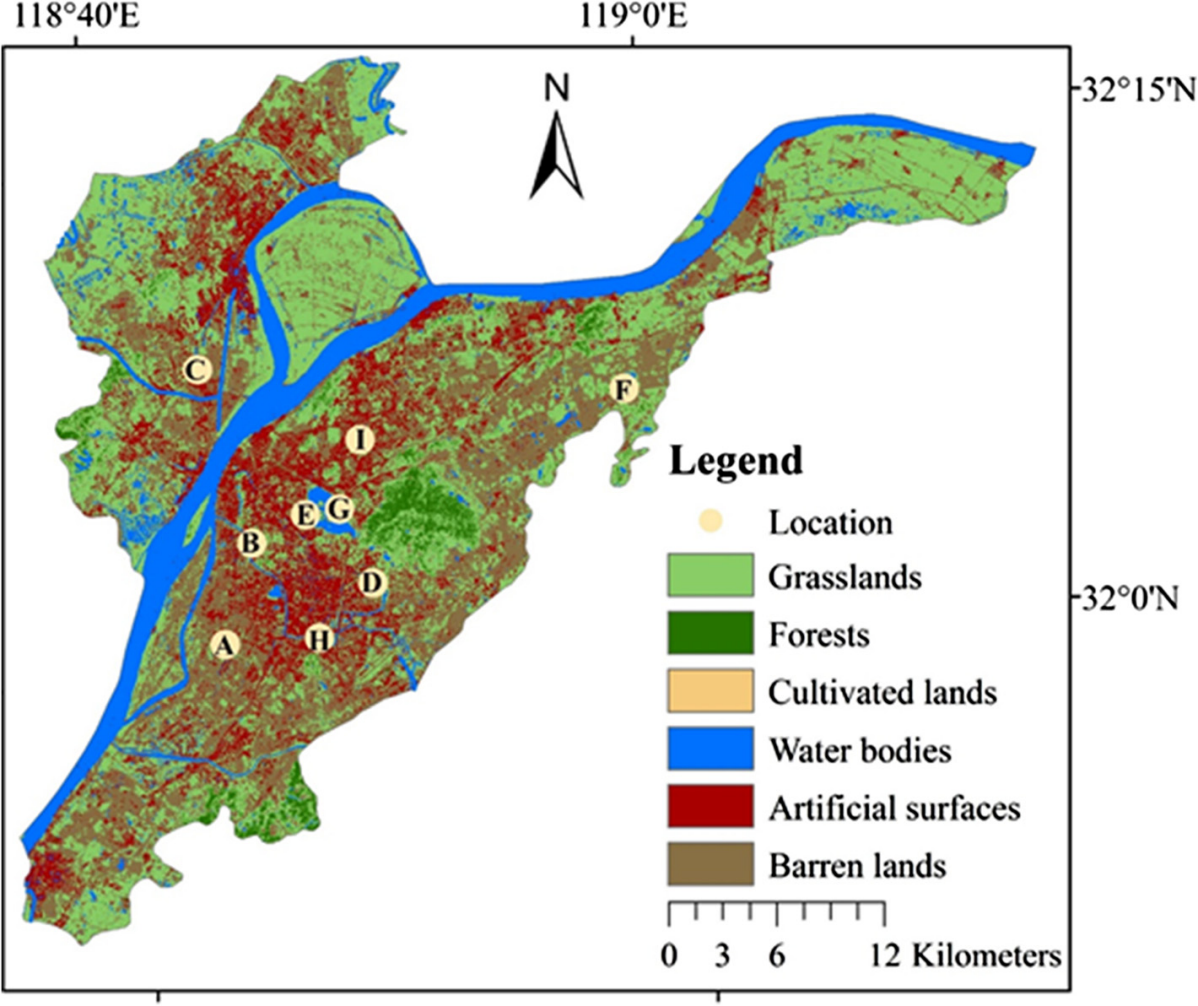
Spatial distribution of the nine air quality monitoring stations in Nanjing, China. The land cover map was downloaded from (http://www.globallandcover.com/GLC30Download/index.aspx). Daily PM_2.5_ concentration (μg m^−3^) was recorded from July 11, 2013 to May 31, 2015 (total = 534 days) at nine stations: (*A*) the Olympic Stadium; (*B*) Caochangmen; (*C*) Pukouqu; (*D*) Ruijinlu; (*E*) Shanxilu; (*F*) Xianlindaxuecheng; (*G*) Xuanwuhu; (*H*) Zhonghuamen; and (*I*) Maigaoqiao

Nanjing (119° E and 32° N) in Jiangsu province of China was used as our study landscape (Fig. 1). Located at the lower reaches of the Yangtze River, Nanjing was the capital of China until 1949 and is now a major industrial center along the river. The Yangtze River, the major inland transportation route in China, dissects the city southwest-northeast direction. The region has a warm temperate climate and is influenced by the East Asian monsoons, with four distinct seasons. The winters are cool and damp while the summer is very hot and humid, which led to Nanjing being named one of the “sThree Furnace-like Cities” within the Yangtze River Basin. The average annual temperature is ~15.5 °C with an annual precipitation of 1060 mm; most rainfalls are between March and August (Liu 1985). Regardless of its large population (>8 million), rapid urban expansion since 1990, and growing industries, Nanjing has kept relatively large portions of forests and grasslands (including lawns) at 3.9 and 24.2 % of the total landscape (5026.14 km^2^), respectively, which together amount to a green cover of 34.5 %. The major industries that emit pollutants and create fugitive dust include automobile and coal (Huang et al. 2006). The number of vehicles in Nanjing was estimated at 2.06 million in 2014.

We recorded daily PM_2.5_ concentration from the CNEMC’s air quality report webpage, which began disseminating public reports in 2013 (http://113.108.142.147:20035/emcpublish/). These reports covered over 190 cities (>1500 sites) and were made available for the public several times a day. However, they were not archived in a comprehensive database for open access. Consequently, we took the report directly from the webpage for all nine stations in Nanjing (http://aqicn.org/city/nanjing/) and built our own database. We calculated the daily mean in our database when several reports per day were available from July 11, 2013, through May 31, 2015 (total = 534 days). This period covered each season twice. The nine stations were not evenly distributed across the city but were aggregated around the downtown area (Fig. 1). Their locations were (A) the Olympic Stadium; (B) Caochangmen; (C) Pukouqu; (D) Ruijinlu; (E) Shanxilu; (F) Xianlindaxuecheng; (G) Xuanwuhu; (H) Zhonghuamen; and (I) Maigaoqiao. Based on the PM_2.5_ concentration and CNEMC standards, each day was categorized as either non-polluted, which ranked either “excellent” (<35 μg m^−3^) or “good” (35–70 μg m^−3^), or as polluted, which ranked as “light” (75–115 μg m^−3^), “intermediate” (115–150 μg m^−3^), “heavy” (150–250 μg m^−3^), or “very heavy” (>250 μg m^−3^) (http://kjs.mep.gov.cn/hjbhbz/bzwb/dqhjbh/jcgfffbz/201203/W020120410332725219541.pdf).

We divided the year into four seasons based on the standards of the Chinese Meteorological Administration to calculate the seasonal mean, minimum, maximum, and standard deviation: spring (March–May), summer (June–August), autumn (September–November), and winter (December–February). Due to the availability of the data (i.e., 534 days), this division of the seasons yielded an uneven number of days among the seasons. However, each season had at least 90 days as the sample size to assure the confidence for calculating the relevant statistics. These statistics of PM_2.5_ concentrations were also calculated for weekdays and weekends because both industrial activities and the use of automobiles—the two largest emission sources of PM_2.5_—may differ (Masetti et al. 2015). The monthly and seasonal mean and the standard error of PM_2.5_ were used to generate the spatially continuous change of PM_2.5_ through an inverse distance weighted (IDW) method. The exponent of distance was set at 12 km, which was the significance of surrounding points on the interpolated value (Bartier and Keller 1996). This spatial interrelation method had more advantages than others, such as Kriging (Rohde and Muller 2015) or the spline method (John et al. 2013). To further explore the spatial variation under extreme high/low PM_2.5_ conditions, we selected December 13, 2013 (classified as “heavy” pollution) and September 9, 2014 (classified as “excellent” condition) to illustrate the spatial changes in PM_2.5_ concentration (Table 1).

**Table 1:** Frequency table of PM_2.5_ concentration by pollution class over the 534-day study period (July 11, 2013–May 31, 2015) in Nanjing, China. Days exceeding PM_2.5_ of 75 μg m^−3^ are considered “polluted,” i.e., higher than the national standard according to the Technical Regulation on Ambient Air Quality Index issued by the Ministry of Environmental Protection of People’s Republic of China (http://kjs.mep.gov.cn/hjbhbz/bzwb/dqhjbh/jcgfffbz/201203/W020120410332725219541.pdf)

Daily PM_2.5_ (μg m^−3^)	Pollution class	No. of days and proportion (%) during the study period				
		Total	Spring	Summer	Autumn	Winter
0–35	Excellent	92 (17.2)	20 (21.7)	32 (34.8)	27 (29.4)	13 (14.1)
35–75	Good	247 (46.3)	84 (34.0)	51 (20.6)	60 (24.3)	52 (21.1)
Sub total		339 (63.5)	104 (30.7)	83 (24.5)	87 (25.6)	65 (19.2)
75–115	Light	115 (21.5)	23 (20.0)	10 (8.7)	38 (33.0)	44 (38.3)
115–150	Intermediate	43 (8.1)	5 (11.6)	2 (4.7)	17 (39.5)	19 (44.2)
150–250	Heavy	28 (5.2)	2 (7.1)	2 (7.1)	4 (14.3)	20 (71.5)
>250	Very heavy	9 (1.7)	0 (0.0)	0 (0.0)	0 (0.0)	9 (100.0)
Sub total		195 (36.5)	30 (15.4)	14 (7.2)	59 (30.2)	92 (47.2)

The global land cover product of GLOBELAND30 (http://www.globallandcover.com/home/Enbackground.aspx) was used to quantify the landscape structure because of its availability to the general scientific community (Chen et al. 2014). There are ten classes in this product: water bodies, wetlands, artificial surfaces, tundra, permanent snow and ice, grasslands, barren lands, cultivated lands, shrublands, and forests. Tundra and permanent snow and ice do not exist in Nanjing. We placed shrublands under the forest class because the landscape is likely composed of lawns with sparse trees; and we placed the small amount of wetlands into the grasslands class, yielding a total of six land cover types: grasslands, forests, cultivated lands, water bodies, artificial surfaces, and barren lands (Fig. 1). Finally, we condensed the categories in this study and merged grasslands, forests, and cultivated lands together to form the green cover class.

The reclassified cover map was imported into ArcGIS to calculate a suite of landscape metrics (~100 indices) using the FRAGSTATS 4.2—a computer software program designed to compute a wide variety of landscape metrics (McGarigal and Marks 1994). Assuming that the potential influence from vegetation existed on PM_2.5_ concentration, it was logical to consider that vegetation near a monitoring station played stronger roles than those far away. In this study, we clipped seven landscapes from the GLOBELAND30 for each of the nine stations with a radius of 0.5, 1.0, 2.0, 3.0, 4.0, 5.0, and 6.0 km (i.e., scale) before the FRAGSTATS was applied to calculate the metrics; this allowed an approximate of 240 tree-height footprint size. Correlation analyses were first performed between the PM_2.5_ concentration and all metrics by month, season, and the entire period to explore the importance of different structural measures at each scale (i.e., different radius). Our preliminary correlation analyses indicated that forest cover, grassland cover, total green cover, and total edge length had high correlations with PM_2.5_ concentration. Consequently, we focused on these metrics when developing empirical models where each metric was log-transferred to assure the normality of residuals. All interactive terms (e.g., green cover × edge length) were included in developing the multivariate models. All statistical analyses and modeling were performed using the SAS9.4 package, including the general linear model (GLM) and the factorial analysis of variance (ANOVA).

## Results

**Fig. 2:**
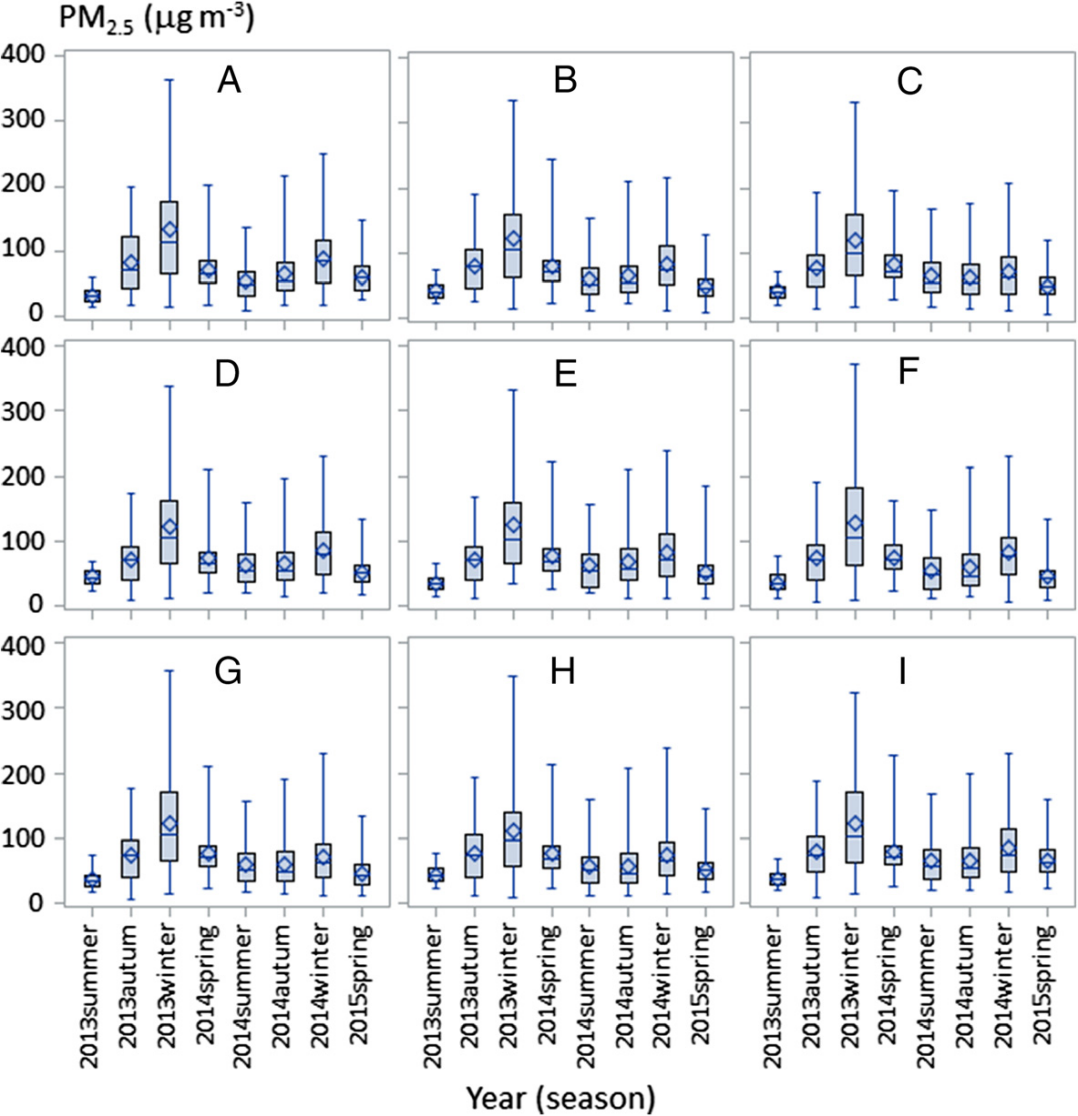
*Boxplots* of seasonal mean PM_2.5_ concentrations at nine monitoring stations (Fig. 1) in Nanjing over the 534-day study period. The nine stations are **a** the Olympic Stadium; **b** Caochangmen; **c** Pukouqu; **d** Ruijinlu; **e** Shanxilu; **f** Xianlindaxuecheng; **g** Xuanwuhu; **h** Zhonghuamen; and **i** Maigaoqiao

The daily average, minimum, and maximum PM_2.5_ concentration of the nine monitoring stations over the 534-day study period was 74.0, 14.2, and 332.0 μg m^−3^, respectively, with an in situ (Xianlindaxuecheng) maximum of 372.0 μg m^−3^ on December 4, 2013, and a minimum of 5.5 μg m^−3^ on September 24, 2013. There appeared to be no significant difference (*P* < 0.05) among the nine stations at daily, monthly, and seasonal scales; however, the average difference varied by ±23.7 μg m^−3^ (~12.7 % of the mean) (Fig. 2). The PM_2.5_ concentration at the Olympic Stadium had the highest deviation from the city mean (13.2 μg m^−3^), whereas Caochangmen had the closest value to the mean (7.6 μg m^−3^ from the mean). Overall, Maigaoqiao had the highest PM_2.5_ concentration (77.2 μg m^−3^), whereas Zhonghuameng had the lowest (69.6 μg m^−3^). Over the 534-day study period, 339 days (63.5 %) were categorized as either “excellent” or “good” conditions according to CNEMC’s standards (Table 1). However, these days were found mostly in the spring (30.7 %), autumn (25.6 %), and summer (24.5 %), with only 19.2 % of the days in the winter. The remaining 195 days (36.5 %) were placed in the “polluted” category, of which 115 days were recorded with a PM_2.5_ concentration of 75–115 μg m^−3^, 43 days (8.1 %) with a PM_2.5_ concentration of 75–115 μg m^−3^, 28 days with a PM_2.5_ concentration of 115–150 μg m^−3^, 28 days with a PM_2.5_ concentration of 150–250 μg m^−3^, and 9 days with a PM_2.5_ concentration >250 μg m^−3^.

**Fig. 3:**
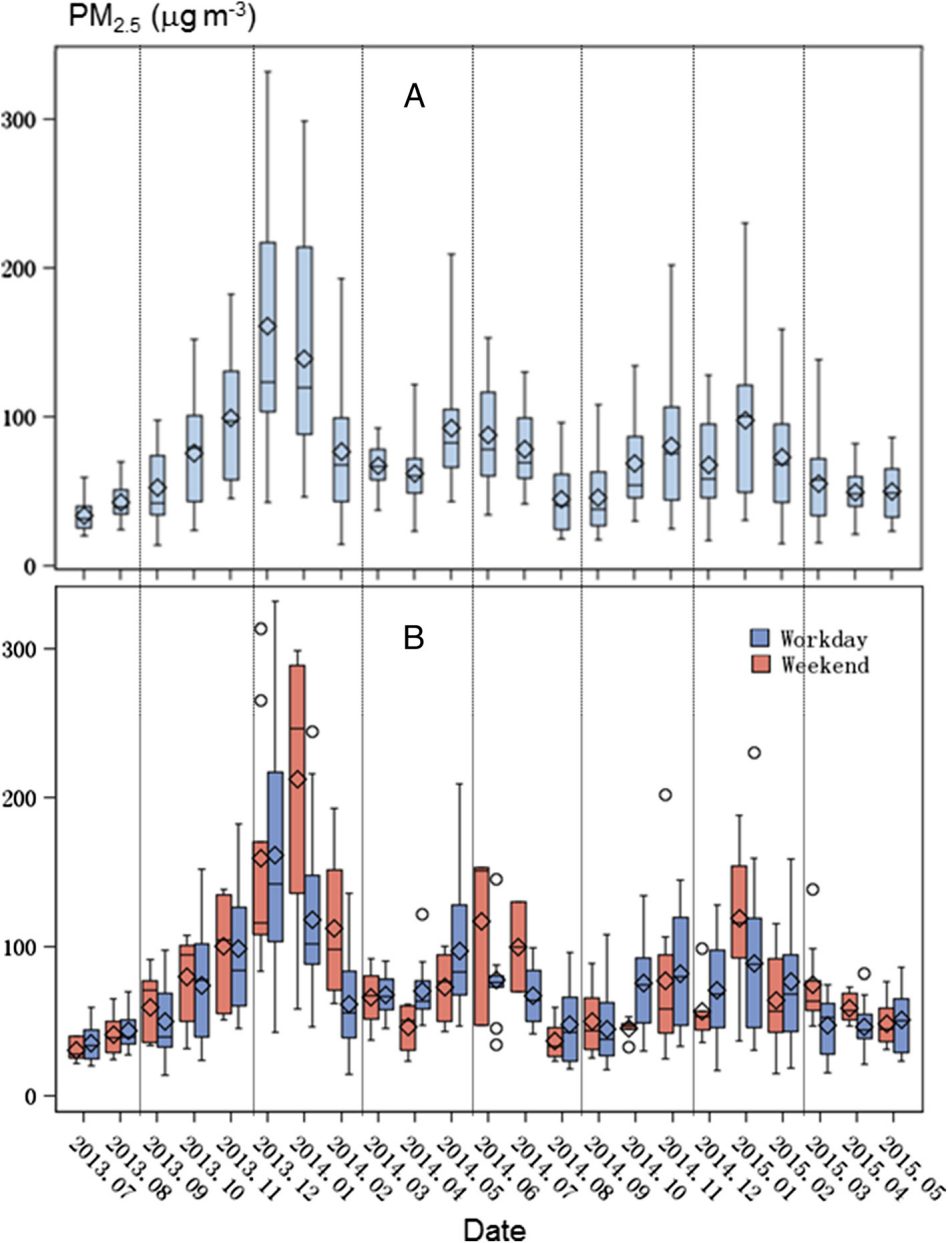
*Boxplots* of monthly mean PM_2.5_ concentration (*n* = 9) in Nanjing over the 534-day study period, including the differences between **a** weekdays (Monday–Friday) and **b** weekends (Saturday and Sunday)

**Fig. 4:**
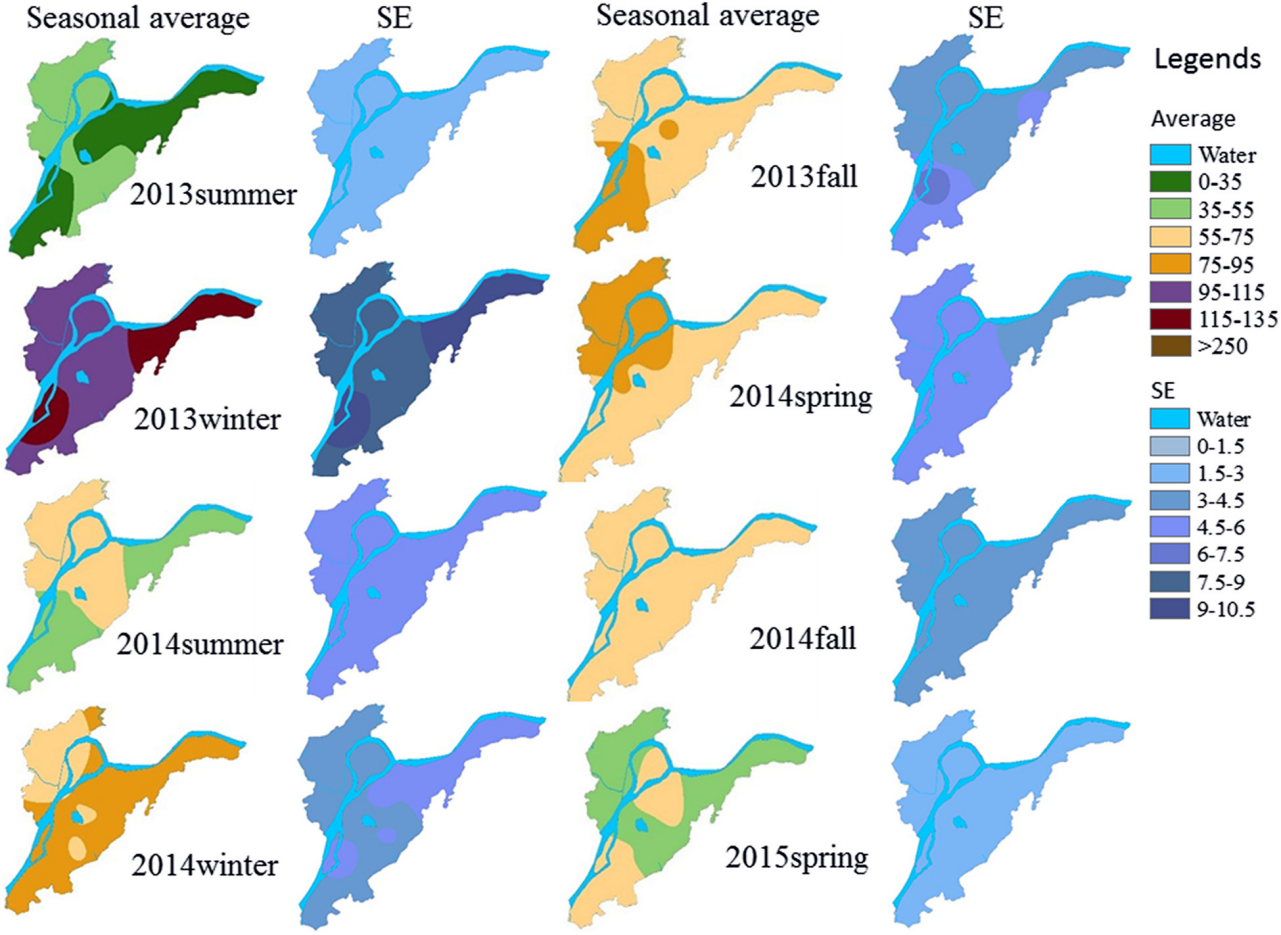
Spatial changes in the mean and variation (standard error (SE)) of PM_2.5_ concentration over four seasons during the study period from July 2013 to May 2015. The inverse distance weighted (IDW) method was used in the spatial interpolations with the exponential distance of 12 and 9 as the number of points

**Fig. 5:**
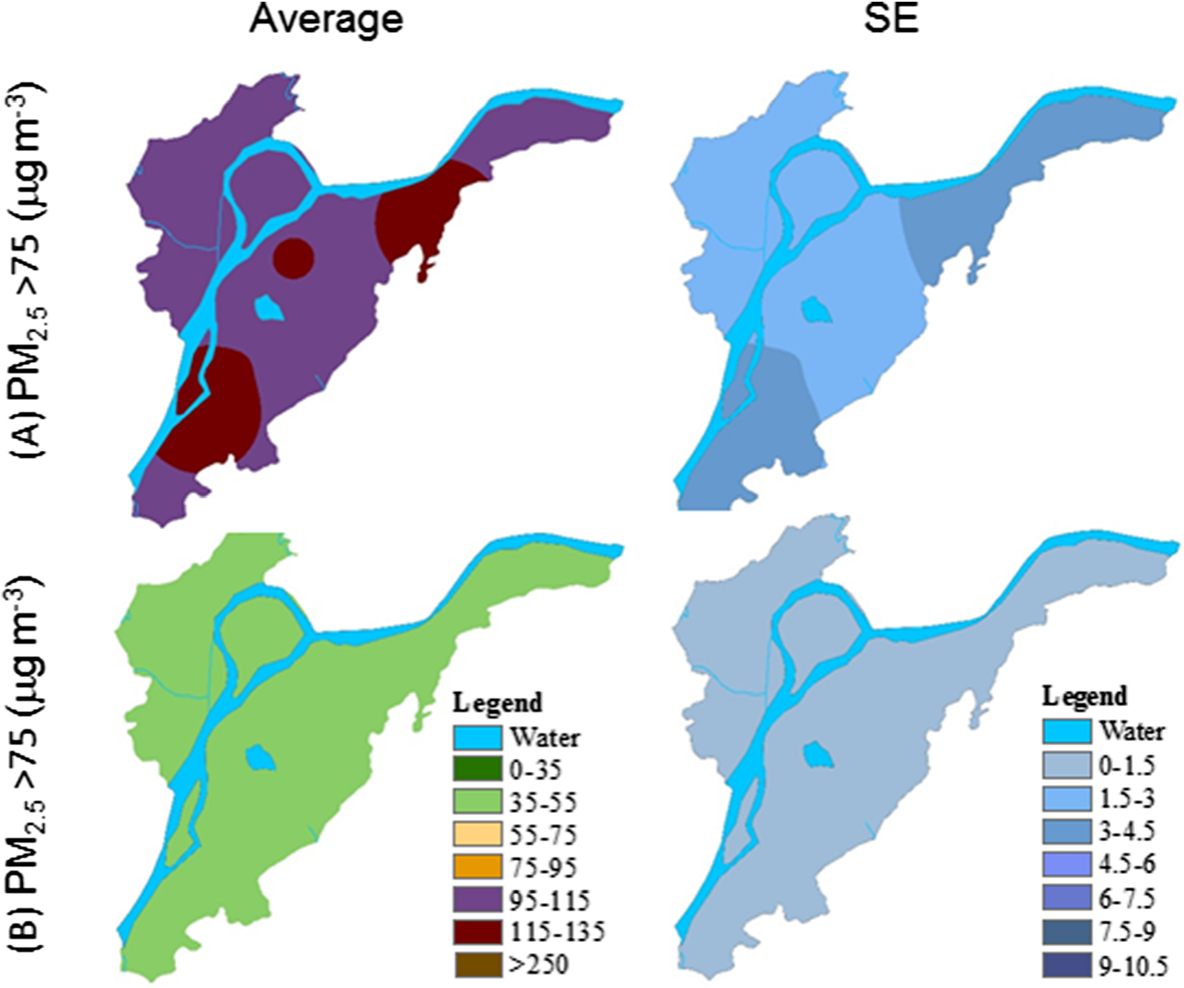
Spatially interpolated mean and variation (standard error (SE)) of PM_2.5_ concentration above/below the 75 μg m^−3^ threshold during the study period from July 2013 to May 2015

Temporally, PM_2.5_ concentration varied greatly at daily to yearly scales (Figs. 2 and 3). Spatially, PM_2.5_ concentrations greater than 75 μg m^−3^ were most frequently found in the east (Maigaoqiao, Xianglindaxuecheng) and the southwest (the Olympic Stadium) (Figs. 4 and 5). High PM_2.5_ concentrations that exceeded CNEMC’s safe standard were recorded predominately during the winter (39.3–100 %), which appeared true for all nine stations (Fig. 2). The days with PM_2.5_ concentrations of >250 μg m^−3^ (“very heavy” pollution) were all found during the two winters. During the winter of 2013–2014, over 50 % of the days had “heavy” pollution (i.e., PM_2.5_ >150 μg m^−3^). Surprisingly, we found that spring had a higher number of unpolluted days than summer and autumn. These seasonal differences in PM_2.5_ concentration appeared in December 2013 and January 2014 when the monthly mean reached 161.1 and 139.5 μg m^−3^, respectively (Fig. 3a). However, the monthly means of the two springs were not always lower than 75 μg m^−3^, while the monthly means of both summers were within this safety level.

We suspected that the pollution level might have been related to emission activities that were different between weekdays and weekends. However, no clear differences existed between the weekdays and weekends during the 23-month study period. For 11 months, the PM_2.5_ concentration was higher during the weekdays than during weekends, which was found in all seasons (Fig. 3b). Additionally, we found that the monthly mean PM_2.5_ for 7 months was substantially higher during the weekdays than the weekends (January 2014, February 2014, June 2014, July 2014, January 2015, March 2015, and April 2015). In comparison, only 3 months (May 2014, August 2014, and December 2014) were found to have higher PM_2.5_ concentrations during the weekends than during the weekdays.

There existed clear spatial distributions and variations in PM_2.5_ across the Nanjing landscape, with distinct patch patterns across seasons (Fig. 4). These patterns were found for all seasons except for autumn of 2014. However, the overall spatial variation, which was measured by the standard error of the mean, was low except in the winter of 2013. In both winters of 2013 and 2014, patches of high PM_2.5_ appeared in the east and southwest of the landscape. The PM_2.5_ concentration in the center (i.e., the downtown area and near the Purple Mountain) and northern (i.e., new development areas) parts were lower than that in the other parts of the landscape, except for the summer and winter of 2014 and spring of 2015. We identified three patches of high PM_2.5_ concentrations that exceeded 75 μg m^−3^ (i.e., the hotspots), with two in the northeast and one in the southwest of Nanjing (Fig. 5a). When the PM_2.5_ was lower than 75 μg m^−3^ (i.e., non-polluted), there were no observable spatial changes or variations (Fig. 5b).

**Fig. 6:**
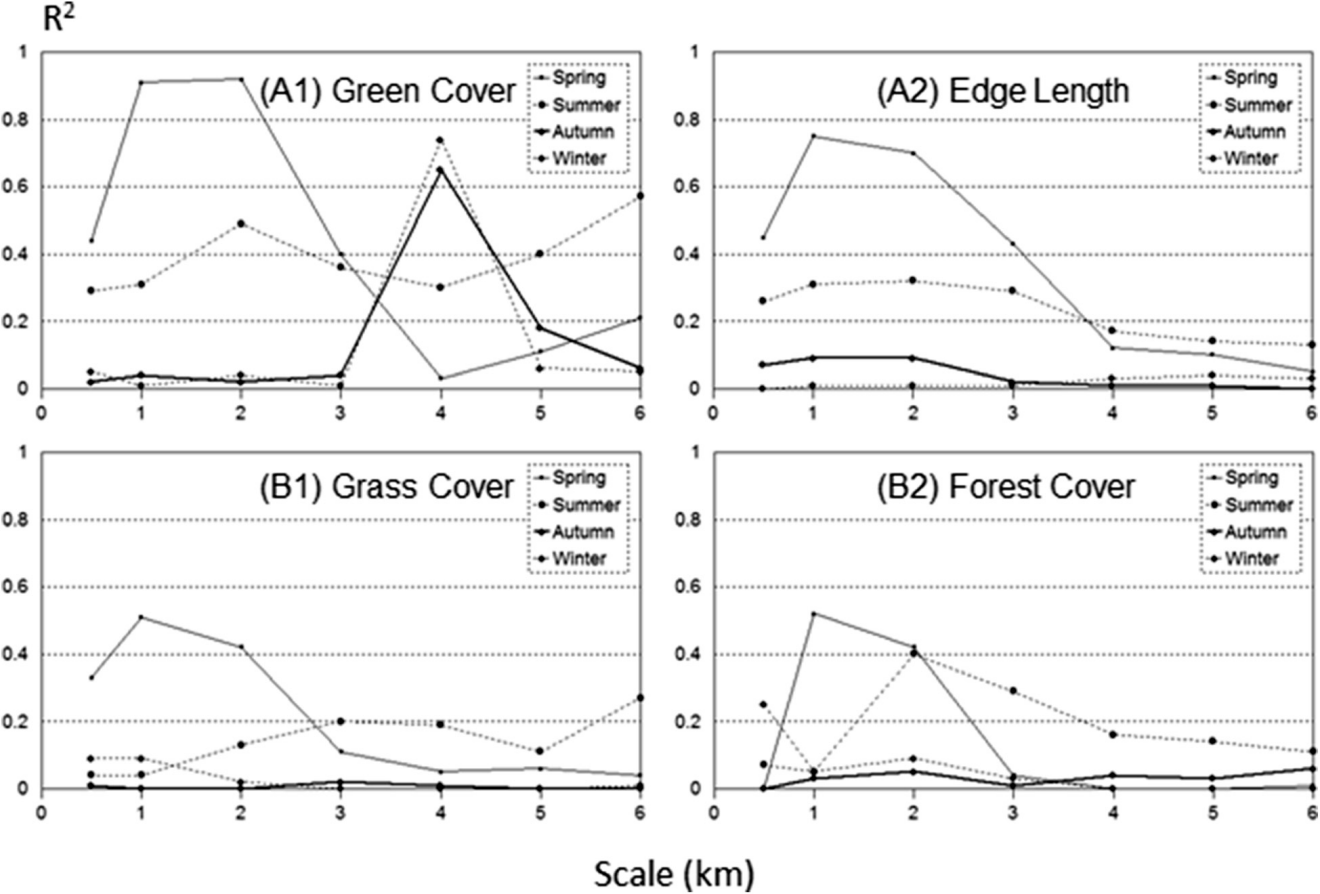
Changes in the correlation coefficient of determination (*R* ^2^) between PM_2.5_ concentration and landscape structure, with scales over four seasons in Nanjing during 2015

Green vegetation had the potential to reduce PM_2.5_ concentration in specific seasons and at some scales (Table 2). Through a simple correlation regression analysis between the PM_2.5_ concentration and all landscape metrics (see the “Methods” section), we found that forest cover, grass cover, total green cover, and total edge length around the green covers were highly correlated with PM_2.5_ concentration (Fig. 6). However, the strength of the correlation varied by scale and by season (Fig. 6). The PM_2.5_ concentration appeared very highly correlated (*R*^2^ > 0.85), with green cover in spring at 1–2 km scales, highly correlated (*R*^2^ > 0.6) in autumn and winter at a 4 km scale, and moderately correlated in summer (*R*^2^ > 0.4) at 2-, 5-, and 6-km scales (Fig. 6a1). For edge length, high and moderate correlations (*R*^2^ > 0.6, *R*^2^~0.35) at scales of 1–3 km were also detected between PM_2.5_ and total edge length during spring and summer, respectively (Fig. 6a2). Surprisingly, only a moderate correlation (*R*^2^ > 0.4) was found with both grass cover and forest cover at scales of 1.0–2.0 km (Fig. 6b1 and b2). No significant correlation was found between PM_2.5_ concentration and total green cover when PM_2.5_ concentration was greater than 75 μg m^−3^ (Table 2).

**Table 2:** Changes in *P* value from simple linear models that predict PM_2.5_ concentration from total green cover, total edge length, grass cover, and forest cover at seven different scales by season from July 11, 2013, through May 31, 2015 in Nanjing, China

Radius (km)	Spring	Summer	Autumn	Winter	Spring	Summer	Autumn	Winter
	Green cover				Edge length			
0.5	0.058	0.656	0.131	0.921	*0.047*	0.486	0.164	0.921
1	*0.005*	0.772	0.092	0.882	*0.003*	0.441	0.120	0.870
2	*0.005*	0.274	*0.014*	0.741	*0.006*	0.437	0.115	0.933
3	*0.039*	0.830	*0.049*	0.890	0.053	0.702	0.133	0.807
4	0.551	0.743	0.218	0.969	0.363	0.776	0.264	0.654
5	0.830	0.567	0.847	0.790	0.405	0.829	0.322	0.602
6	0.842	0.589	0.928	0.674	0.577	0.987	0.331	0.650
	Grassland cover				Forest cover			
0.5	0.107	0.764	0.589	0.429	0.936	0.914	0.506	0.171
1	*0.041*	0.972	0.628	0.433	*0.028*	0.653	0.583	0.560
2	0.058	0.956	0.336	0.705	0.061	0.559	0.066	0.443
3	0.378	0.703	0.232	0.898	0.590	0.776	0.133	0.659
4	0.555	0.835	0.243	0.941	0.958	0.625	0.286	0.940
5	0.511	0.934	0.206	0.902	0.906	0.640	0.326	0.996
6	0.585	0.906	0.151	0.866	0.814	0.510	0.381	0.984
	Green cover (PM_2.5_ ≥ 75 ug·m^−3^)				Green cover (PM_2.5_ < 75 ug·m^−3^)			
0.5	0.856	0.531	0.379	0.968	*0.003*	0.275	0.227	0.336
1	0.445	0.391	0.260	0.992	*0.002*	0.264	0.493	0.385
2	0.315	0.292	0.176	0.880	*0.001*	0.174	0.470	0.277
3	0.519	0.252	0.102	0.988	*0.011*	0.438	0.672	0.253
4	0.704	0.377	0.193	0.784	0.213	0.457	0.390	0.109
5	0.799	0.379	0.163	0.567	0.430	0.554	0.905	0.176
6	0.834	0.394	0.202	0.513	0.793	0.342	0.508	0.319

Single and multiple variables were used to develop empirical models to predict PM_2.5_ concentrations for each of the five significant scales of the corresponding seasons (Table 3). Among all the models, log(green cover) × log(edge length) as well as log(green cover) were found to be significant (Table 3). The first model was proficient in predicting spring PM_2.5_ concentration at a 1.0–2.0 km scale, summer at a 4.0 km scale, autumn at a 6.0 km scale, and winter at a 4.0 km scale. When log(green cover) was used as the single variable, the model seemed proficient at the same season (scale). Regardless of the models’ significance, the extreme low values of the correlation coefficient of determination (*R*^2^) forced us to reject the model when predicting PM_2.5_ concentration in summer, autumn, and winter. For spring, edge length alone can be used to predict PM_2.5_ with a confidence of 83 and 82 % at 1.0 and 2.0 km scale, respectively. Inclusion of green cover did not improve our predictive capability (Table 4).

**Table 3:** *P* values of the two selected models for predicting PM_2.5_ concentration from total green cover (%) and total edge length (km) at seven scales by season in Nanjing from July 11, 2013 through May 31, 2015

Radius (km)	Spring	Summer	Autumn	Winter	Spring	Summer	Autumn	Winter
	Log(green cover)*log(edge length)				Log(green cover)			
0.5	0.462	0.810	0.586	0.597	0.294	0.852	0.431	0.611
1	*0.009*	0.651	0.684	0.845	*0.050*	0.679	0.911	0.842
2	*0.003*	0.788	0.502	0.891	*0.020*	0.774	0.850	0.968
3	0.385	0.800	0.412	0.903	0.562	0.744	0.582	0.880
4	0.976	*0.018*	0.398	*0.006*	0.957	*0.017*	0.364	*0.006*
5	0.432	0.367	0.091	0.667	0.457	0.327	0.097	0.721
6	0.254	0.695	*0.031*	0.985	0.251	0.637	*0.031*	0.930

**Table 4:** Estimated regression coefficients in five significant models predicting PM_2.5_ concentration (Table 3) from log-transformed independent variables of total green cover (%) and total edge length (TE, km)

Season (scale)	Log(green cover)*log(edge length)			Log(edge length)		
	Intercept	Slope	*R* ^2^	Intercept	Slope	*R* ^2^
Spring (1.0 km)	56.93	3.57	*0.82*	50.24	−10.75	*0.83*
Spring (2.0 km)	56.05	4.50	*0.79*	48.71	−12.28	*0.82*
Summer (4.0 km)	50.79	−0.78	0.03	49.06	−0.41	0.01
Winter (4.0 km)	100.97	−0.09	0.01	104.37	3.01	0.02
Autumn (6.0 km)	69.59	0.46	0.01	65.49	−4.64	0.12

## Discussion

Air pollution worldwide has been a consequence of increasing energy consumption, urban expansion, population growth, and vehicle use, which have compounded due to relatively low investments in emission control and processing technology. Extreme pollution levels have been reported in many cities, such as Los Angeles (USA), London (UK), Hong Kong (China), Milano (Italy), and now Delhi (India), Beijing (China), Mexico City (Mexico), Ulaanbaatar (Mongolia), and others in developing countries (Rodrıguez et al. 2004; Apte et al. 2015; Fan et al. 2016). In Nanjing, the government issued its first-ever red alert for poor air quality on December 6, 2013, due to levels of harmful PM_2.5_ reaching higher than 300 μg m^−3^ and lasting for more than 12 h, which reduced visibility to 1 km. This level, however, is much lower than that found in neighboring Shanghai, where a record of PM_2.5_ greater than 1000 μg m^−3^ was reported on March 28, 2014. Earlier in Shenyang, located in Northeast China, PM_2.5_ was detected at a record of 1400 μg m^−3^—50 times above what the World Health Organization (WHO) recommends as safe—on November 9, 2015, marking it the highest pollution on record since China began monitoring air quality in 2013. Even as this manuscript was written, Beijing issued a “Red Warning” twice in December 2015 due to the dangerous PM_2.5_ levels and required that all schools close and residents stay at home.

Smog is now an infamous term frequently used in education, policymaking, and public communities, as well as pressing issues concerning science. Of all the pollutants, PM_2.5_ is the most problematic because of its complex and dangerous species composition and size. In London, a recent study found that ~9500 people die each year because of air pollution (Walton et al. 2015; Zupancic et al. 2015). Apte et al. (2015) estimated that ambient PM_2.5_ is responsible for about 750,000 deaths annually worldwide and claimed that even a 20–30 % reduction in the average PM_2.5_ levels over the next 15 years would merely offset the increase of PM_2.5_-attributed deaths in aging populations. In pursuit of sustainable urban development, societies have begun seriously seeking innovative technology, emission controls, green space enhancement (i.e., nature-based solutions), alternative policies, and other solutions (Janhäll 2015).

Green spaces in urban landscapes are increasingly recognized and promoted because of their crucial roles in urban ecosystems and human health, such as air purification, carbon sequestration, reduction of urban heat islands, and provision of recreational spaces (Chiesura 2004; Tzoulas et al. 2007; Sanesi et al. 2009; Tian et al. 2014; Wolch et al. 2014). They are particularly effective in “capturing” pollutants through their abundant surface areas, such as leaves and bark (Nowak et al. 2013; Janhäll 2015). Our results are consistent with previous experiments in Nanjing (Huang et al. 2002) and elsewhere (Nowak et al. 2013; Wu et al. 2015), while our results on the negative correlation between green cover and PM_2.5_ concentration (Fig. 6, Table 4) support our hypothesis. However, the empirical relationship varies greatly by season, the degree of PM_2.5_ level, and the distance from the point of concern (e.g., emission source, high population concentration) (Table 2).

At first, vegetation cover of forest, grassland, and total green space seemed significant in reducing PM_2.5_ concentration only within 1.0–3.0 km of a study point, not within the first 1.0 km or beyond 3.0 km. Yet, edge density within also 2.0 km appeared significant (Table 2). In other words, the PM_2.5_ concentration of a point in the landscape can be significantly modified by dispersing smaller vegetation patches within 2.0 km as well as by the total green cover within 1.0–3.0 km. These interesting results need to be explored through the development of an aerodynamic footprint model (e.g., Schmid 1994), source-sink transportation, and large eddy simulations in the future. Our results can be used when managing green spaces in specific locations. Yet, if our goal is to manage the entire landscape, then increasing total cover—especially forest cover—and edge density are two plausible recommendations. Another outcome from these findings is that future green space promotions are possible through targeting specific emission sources (i.e., effective reduction), population sizes, and distribution (i.e., benefits to people). More so, future green space management may consider more evergreen species, especially within the surrounding 2–3 km vicinity of a major emission source and other locations with high populations. Nevertheless, caution should be taken when extrapolating these results to other urban landscapes in different climate zones because of high contrasts in both landscape structure and atmospheric movement (Hao et al. 2015). Finally, the empirical models were developed with existing PM_2.5_ data and a static landscape. Because both dependent and independent variables can be different in the future, mechanistically based modeling is urgently needed.

It also appeared that the significant pollution-removal function of green spaces in Nanjing existed in spring and at some degrees in autumn (Table 2), with edges showing no obvious correlation in either summer or winter (Fig. 6). These variations among the seasons can be potentially coupled with (1) rapid changes in vegetation leaves in spring and autumn and a stable amount of leaves in the summer and winter; (2) absorption of PM_2.5_ by rain water; and (3) high precipitation in the spring monsoon, which can wash away intercepted pollutants on a plant’s surface and increase wet deposition, and to some degree, dry deposition. As air moves through a landscape, pollutants are deposited on surfaces and later fall (i.e., dry deposition) or wash away to the ground during precipitation events. This process will result in higher pollutants within forests and other green spaces (Xu et al. 2002; Wu et al. 2008). Janhäll (2015) concluded that the “filtration vegetation barriers have to be dense enough to offer a large deposition surface area and porous enough to allow penetration.” The differences in canopy cover and the high porosity across the edges might provide partial explanations for the insignificant influences in the summer and the winter. With longer edges between vegetation patches and neighboring open spaces, vegetation can potentially capture more pollutants carried through horizontal advection, resulting in higher deposition near the edges (Wu et al. 2015). Additionally, the relatively humid environment (Chen et al. 1993) has a higher capacity in absorbing pollutants (i.e., wet deposition) (Nowak et al. 2013; Wu et al. 2015). Because PM_2.5_ is small in size, “capturing” them might be more effective than with other, larger particulates. Consequently, denser, taller vegetation (e.g., forests; Chen et al. 1992), higher green space coverage, and more heterogeneous vegetation patches (i.e., higher edge density) can produce stronger effects on PM_2.5_ concentration.

Lastly, the role of green spaces in PM_2.5_ concentration reduction varies by pollution level. A major finding of this study was the non-significant correlation between green cover and PM_2.5_ concentration when the concentration was >75 μg m^−3^ (Table 2). This suggests that the relative contribution of green spaces in PM_2.5_ reduction seemed low during high pollution conditions. One should not devalue the importance of green spaces when interpreting these results but instead highlight the need for field experiments to separate the contributions of vegetation among all pollution contributors (i.e., emission sources, dispersions, e.g., Chen et al. 2015). Unfortunately, such high conditions are mostly found in winter when vegetation cover is the lowest—a phenomenon found in most temperate cities (Vecchi et al. 2004; Giugliano et al. 2005; Han et al. 2015).

The underlining processes regulating PM_2.5_ concentrations in Nanjing (and within many cities) are complex and may include excessive emission from factories, rapid increase in automobiles, intensified urbanization, reduction of green space, stabilization in air conditions, development of heat islands, and natural meteorological conditions (Huang et al. 2002; He 2010; Janhäll 2015; Zhang and Cao 2015). For example, the unusual higher-than-average precipitation in late February and March of 2014 may be the primary reason for its low PM_2.5_ concentration (Fig. 3). During the short period of this study, the Youth Olympics Games were held in Nanjing during August 16–28, 2014. Intensified last-minute constructions prior to the games might have been responsible for the high PM_2.5_ concentration in May–July 2014, while the low concentration in August (Fig. 3) may have been a result of the government’s efforts to close many factories and traffic controls several days before and during the games. Consequently, the dynamics and the level of PM_2.5_ in the summer of 2014 were dramatically different from that in the summer of 2013. A similar policy was also implemented during December 2014 when a major memorial service was held for the Nanjing Massacre during World War II. This also resulted in lower PM_2.5_ levels for both the month as well as for the winter of 2014–2015, which was coupled with very unstable atmospheric conditions (Liu et al. 2005). In this study, we examined the dynamics of PM_2.5_ concentrations and their potential effects of green vegetation but excluded many other driving mechanisms such as the above actions, as well as the climatic influences—a major variable determining the transportation and deposition of all pollutants (Liu et al. 2005).

Our findings may not be applicable for other pollutants, suggesting that additional efforts are needed to expand this study to other pollution species in the future. Additionally, our spatiotemporal analyses were based on a limited number of static green spaces, landscape, and available data from CNEMC—as only nine stations were aggregated in the city center (Fig. 1) during a 1.5-year study period. As more information becomes available, one needs to include detailed vegetation characteristics, which are collected through ground measurements and remote sensing, into spatially explicit models in order to understand the roles of green spaces PM_2.5_ concentration reduction. Ultimately, comprehensive experiments and modeling investigations are needed so that all drivers (physical and anthropogenic) in the sources, sinks, transportation, and in the decomposition of pollutants are included as a scientific base for effective planning and management of green spaces in Nanjing and elsewhere.

## Conclusions

In battling the increasing intensity and frequency of air pollution in urban landscapes, daily PM_2.5_ concentration was manually collected from the CNEMC webpage to quantify the spatiotemporal changes of PM_2.5_—the most dangerous pollutant affecting urban dwellings. The spatiotemporal changes of green spaces were further analyzed at daily, weekly, monthly, and seasonal scales. We found that PM_2.5_ concentrations varied by time and space. Over time, great seasonal differences existed, with winters showing the highest concentration. However, the temporal variations were interrupted by physical (e.g., the intensive monsoon in the spring of 2014) and human activities (e.g., the Youth Olympics Games in August 2014). Across the Nanjing landscape, the east and southwest have had the highest pollution levels. The non-significant correlation between green cover and PM_2.5_ concentration was found when its concentration was >75 μg m^−3^. Total edge length within 2 km of a point was significantly related to the low PM_2.5_ concentration. More importantly, we found that forest cover, grassland cover, total green cover, and total edge length within the 1–3 km vicinities of the monitoring station played significant roles in reducing the PM_2.5_ concentration, particularly in spring. Although the empirical models seemed significant for spring only, one should not devalue the importance of green vegetation in other seasons; instead, one should understand that regulations are complicated by stable vegetation characteristics and different meteorological conditions and human activities.
